# Vision retention in early versus delayed glaucoma surgical intervention in patients with Boston Keratoprosthesis type 1

**DOI:** 10.1371/journal.pone.0182190

**Published:** 2017-08-04

**Authors:** Mark Lin, Anand Bhatt, Asghar Haider, Grace Kim, Marjan Farid, Mason Schmutz, Sameh Mosaed

**Affiliations:** University of California, Irvine, Department of Ophthalmology, Irvine, California, United States of America; Massachusetts Eye & Ear Infirmary, Harvard Medical School, UNITED STATES

## Abstract

**Importance:**

The loss of vision following Boston Keratoprosthesis (BKPro) surgery due to glaucoma occurs at a high frequency as diagnosis and management of glaucoma after this procedure pose challenges.

**Objective:**

To compare visual outcomes in patients undergoing Boston Keratoprosthesis surgery with and without prior or concurrent glaucoma surgery.

**Design, setting, and participants:**

This is a retrospective, observational cohort study of patients who underwent Boston Type I Keratoprosthesis surgery. 19 eyes of 18 patients who had undergone BKPro and met the inclusion criteria were identified. Twelve eyes received BKPro with prior or concurrent glaucoma surgery (Group 1), and seven eyes were identified undergoing BKPro surgery without prior or concurrent glaucoma surgery (Group 2).

**Main outcomes and measures:**

Main outcome included best corrected visual acuity at each follow up.

**Results:**

In Group 1, mean best corrected visual acuity (BCVA) within a year of BKPro surgery was 20/100 (range 20/40 to Count Fingers (CF); n = 12) and mean BCVA at 1 year from BKPro surgery was 20/115 (range 20/30 to CF; n = 12). 7 out of 12 patients retained or had improved BCVA at 1 year follow up after BKPro implantation, and 5 out of 12 patients had mild BCVA worsening. In Group 2, the mean BCVA within a year of BKPro surgery was 20/140 (ranging from 20/25 to hand motion vision (HM); n = 7) and mean BCVA at 1 year from BKPro surgery was Count Fingers (range 20/60 to Light Perception (LP); n = 6). 4 out of 6 patients lost significant vision at one year after BKPro.

**Conclusions and relevance:**

BKPro patients with early glaucoma surgical intervention retained vision significantly better compared to patients with late or no intervention. Our preliminary findings support the recommendation for concurrent or pre-emptive glaucoma surgical intervention in patients undergoing BKPro implantation.

## Introduction

The restoration of a clear visual axis in patients who suffer from corneal blindness can present challenges in those who are also poor candidates for traditional corneal transplantation techniques. These patients often have failed multiple prior penetrating keratoplasties, have ocular surface scarring due to trauma or autoimmune disease, and/or have limbal stem cell deficiencies. The Boston Keratoprosthesis (BKPro) is a prosthetic corneal device that offers an option to restore vision in this cohort of patients. The initial technique was FDA approved for use in 1992 and has evolved to reduce many of the initial shortcomings. This has resulted in more frequent use and greater success in restoring vision in those who are poor candidates for penetrating keratoplasty.

The design of the BKPro consists of 3 main components. There is a corneal graft, which is enveloped between a central optical polymethyl methacrylate (PMMA) button and a posterior plate. This design allows a fixed clear central visual axis with peripheral corneal graft tectonic support to suture to the globe. Although surgeon experience has resulted in improved outcomes and more successful adoption of this technique, there remain several frequently encountered post-operative challenges. These challenges include the development of retroprosthetic membranes [1], a high incidence of endophthalmitis [2] and a significant rate of vision loss from uncontrolled glaucoma [3–5]. Lifelong topical antibiotic prophylaxis and YAG laser membranectomy can be used to address the former two concerns, but the latter has remained a challenge. The typical mechanism of this glaucoma is the synechial closure of the angle following repeat corneal transplantation [6]. Many of these patients have pre-existing angle closure form prior penetrating keratoplasty in addition to ultrastructural changes to any remaining accessible trabecular meshwork tissue from chronic topical steroid usage.

Patients undergoing traditional penetrating keratoplasty are already at increased risk of developing glaucoma due to angle closure or as a result of steroid induced elevation of intraocular pressure. Traditionally, glaucoma after penetrating keratoplasty can be identified through measurement of intraocular pressure and measurement of visual field and treated with traditional methods such as topical medications or glaucoma filtering surgery. Patients undergoing prosthetic corneal transplantation with BKPro may be at the same or greater risk of developing glaucomatous disease after surgery [3–5], however diagnosis and treatment can be a much greater challenge. The most common methods of intraocular pressure (IOP) measurement are dependent on the biomechanical properties of human corneal tissue. After BKPro, however, the replacement of host or human graft cornea with the central corneal button PMMA material precludes accurate IOP measurement. Examiners currently estimate IOP by digital scleral or trans-palpebral palpation, which are notoriously unreliable.

Due to this imprecise measurement of IOP, many patients who do develop glaucoma after BKPro suffer glaucomatous vision loss as a result of delayed diagnosis. Even when elevated IOP is identified postoperatively, the success of topical medications in lowering IOP is challenging to assess and other traditional methods of glaucoma monitoring such as visual field testing and optic nerve imaging are not reliable. Furthermore, should glaucoma surgery be indicated after BKPro, the anatomical restrictions from limited anterior chamber visualization make it preferable to adopt a pars plana implantation of a glaucoma filtering implant with pars plana vitrectomy. Given the frequency of vision loss after BKPro, glaucoma surgery may be of benefit when planned concurrently with BKPro as a measure to prevent vision loss from uncontrolled IOP. In this study, we aim to investigate the relationship between vision retention and early versus late surgical glaucoma intervention in patients with type 1 Bkpro.

## Methods

This is a retrospective, observational cohort study of all patients who underwent Boston Type I Keratoprosthesis surgery between the dates of January 1, 2006 through December 31, 2016 at the University of California, Irvine Ophthalmology Clinic (Orange, CA) and Gavin Herbert Eye Institute (Irvine, CA). All Boston type I Keratoprothesis were performed by a single surgeon and identified using CPT codes for Keratoprosthesis surgery. Institutional Review Board Approval at UC Irvine was obtained for this review, and a waiver for patient consent was granted as this was a retrospective review. 34 eyes of 22 patients were initially identified who had undergone BKPro. A minimum of six months of follow-up was required for inclusion in the study. Eyes that required repeated BKPro, retinal detachment repair, or enucleation during the follow-up period were excluded from the study. Additionally, eyes that had severe retroprosthetic membrane formation, stromal melt, or infectious keratitis that required explanation of BKPro or placement of a new BKPro during the follow-up period were excluded. After applying inclusion and exclusion criteria, 19 eyes of 18 patients were included in this review, and were separated into two groups. Group 1 underwent glaucoma surgery at the time of BKPro or within 3 months of BKPro, and the other group either did not undergo glaucoma surgery or underwent surgery later than 3 months postoperatively. Primary outcome was BCVA at 6 (± 1) months, 12 (± 2) months, 24 (± 2) months, and 36 (± 2) months post-BKPro. BCVA following visual acuity stabilization within 12 months post-BKPro was also measured. Visual Acuity was initially measured with the Snellen chart then converted to a continuous Logarithm of the Minimal Angle of Resolution (Log MAR) units for quantitative analysis. For non-numerical values, the conversions are as follows: Count Fingers = 1.7, Hand Motion = 2.0, Light Perception = 2.3, and No Light Perception (NLP) = 3.0. Each patient’s age, gender, previous surgeries, pre-operative number of glaucoma medications and pre-operative IOP, as measured with applanation or indentation tonometry, were recorded as part of baseline characteristics (Table 1). Original dataset is provided as supporting information (S1 Table).

**Table 1:** Baseline characteristics of patients with Boston type I Keratoprosthesis with or without prior or concurrent glaucoma surgical intervention.

	Group 1 (n = 12)	Group 2 (n = 7)	p-value*
Baseline Demographics^†^			
Mean Age (year ± SD) at time of Kpro	53.3 ± 19.3	60.1 ± 15.4	0.41
Female Gender, no. (%)	4/11 (36%)	3/7 (43%)	1.00
Mean follow-up time (month ± SD)	38.0 ± 16.0	51.4 ± 15.0	0.09
Indication for Kpro surgery			
Graft/KPro failure	12 (100%)	7 (100%)	
Steven-Johnson Syndrome, no. (%)	2 (17%)	2 (29%)	0.60
Chemical Injury, no. (%)	1 (8%)	3 (43%)	0.12
Corneal Decompensation, no. (%)	2 (17%)	1 (14%)	1.00
Aniridia, no. (%)	2 (17%)	0	0.51
Infectious Keratitis, no. (%)	1 (8%)	1 (14%)	1.00
Trauma, no. (%)	4 (33%)	0	0.25
Initial Kpro surgery^‡^, no. (%)	10 (83%)	5 (71%)	0.60
Preoperative glaucoma history			
Mean IOP (mmHg ± SD)	15.6 ± 5.3	15.4 ± 3.8	0.62
History of glaucoma medications, no. (%)	10 (83%)	5 (71%)	0.60
Mean number of glaucoma medications ± SD	2.3 ± 1.8	1.3 ± 1.5	0.27
Prior glaucoma surgery			
Trabeculectomy, no. (%)	2 (17%)	-	
Glaucoma drainage implant, no. (%)	7 (58%)	-	
Concurrent glaucoma drainage implant, no. (%)	4 (33%)	-	

* Fisher exact test used to compare categorical variables between Group 1 and Group 2; Students t-test used to compare mean values between Group 1 and Group 2 assuming unequal variance. Statistical significance is defined as p < 0.05.

^†^ values calculated per patient (11 in group 1 and six in group 2).

^‡^ denotes number of eyes that did not receive prior Kpro surgeries.

Kpro = Boston type I Keratoprosthesis; Group 1 = patients who had glaucoma surgery prior to or concurrently with Kpro; Group 2 = patients with no history of glaucoma surgery or after 3 months post-Kpro; n = number of eyes; SD = standard deviation.

Reliable visual fields, optic disc imaging, retinal nerve fiber layer analysis, or objective post-op IOP measurements were unavailable for each patient at consistent time points post-operatively to be meaningfully compared between the groups, and thus were not reported.

Continuous variables were expressed and tabulated as mean values with corresponding standard deviations. Standard error of mean was represented in graphical form. Categorical values were expressed as discrete integers, frequencies, and/or percentages. Comparison of baseline characteristic means between the two groups was achieved with independent Student’s *t*-test assuming unequal variance, and comparison of mean visual acuity in LogMAR was achieved with the Mann-Whitney *U* nonparametric test. Differences among categorical values between the groups were assessed using Fisher’s exact test (2-tailed). Statistical significance was defined as *p* < 0.05 for all tests.

## Results

Nineteen eyes of 18 patients who underwent Boston Type I Keratoprosthesis implantation meeting the inclusion criteria were identified. Of these 19 eyes in the study, there were no documented cases of endophthalmitis or vitritis during the follow-up period. Twelve eyes of 11 patients received prior glaucoma surgery (n = 8) or glaucoma drainage implants concurrently (n = 4) with BKPro (Group 1). Of the remaining seven eyes in seven patients, six had never received any glaucoma surgery, and one patient received glaucoma surgery 2.5 year after BKPro (Group 2). Of note, two patients in Group 2 received cyclophotocoagulation six months and two years after BKPro, respectively. Table 1 summarizes baseline characteristics of the eyes included in the study. The mean age at time of BKPro implantation for Group 1 was 53.3 years (range of 31–81) versus 60.1 years (range 39–80) for Group 2. All eyes had previous corneal graft failures or failed BKPro prior to BKPro implantation, with the most common reason for initial corneal surgery being ocular trauma (four of twelve eyes) for Group 1, and chemical injury (three of seven eyes) for Group 2. At the start of study period, 10 of 12 eyes in Group 1 had first time BKPro surgeries, versus five of seven eyes in Group 2. Ten of 12 patients in Group 1 had glaucoma medication use prior to BKPro (mean number of glaucoma medications = 2.3) versus five of seven patients in Group 2 (mean number of glaucoma medications = 1.3). Mean follow-up time was 38 months for Group 1, and 51 months for Group 2.

**Fig 1:**
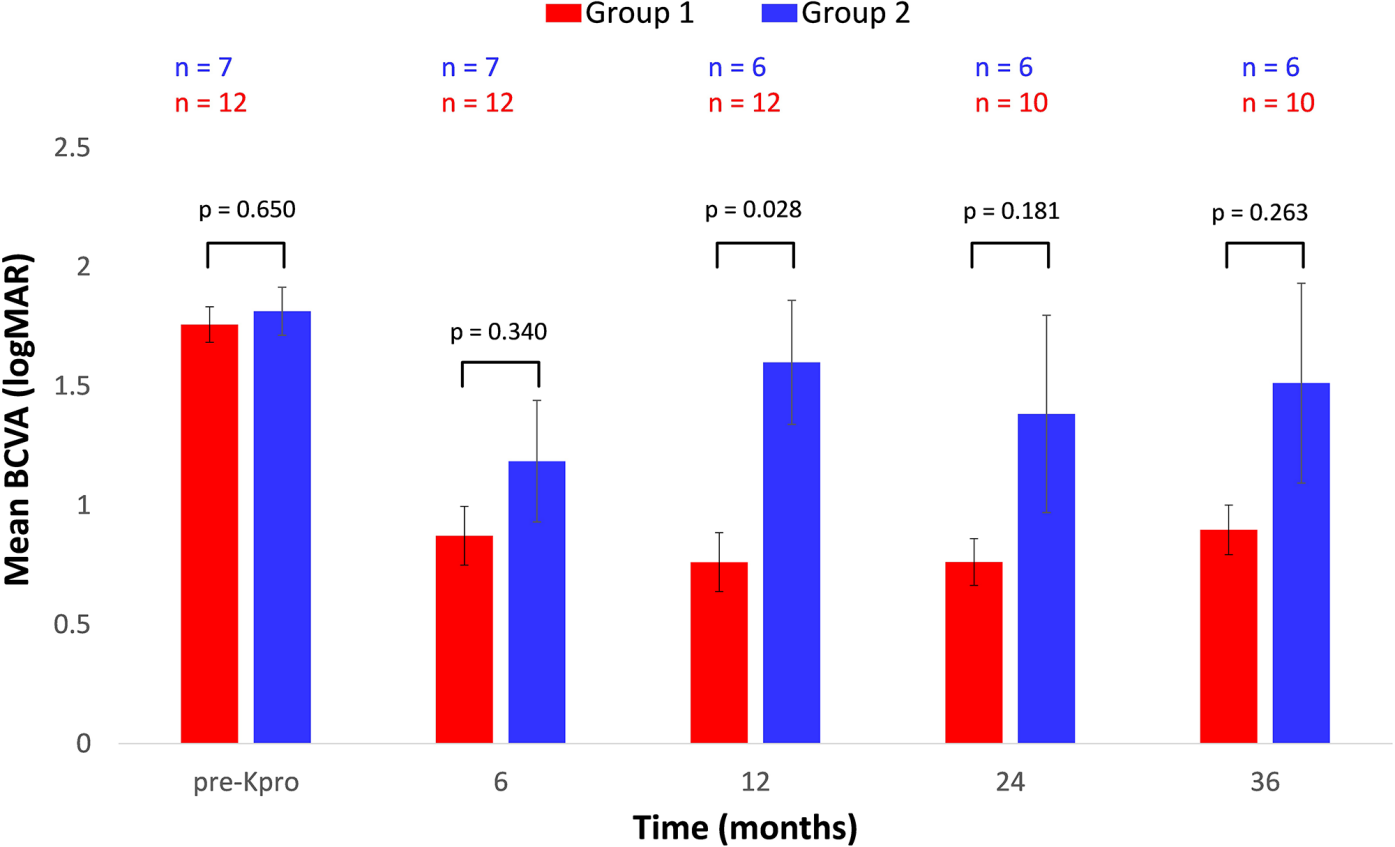
Mean best corrected visual acuity (BCVA) before Keratoprosthesis (pre-Kpro) and at several time points post-KPro implantation for patients with or without prior or concurrent glaucoma surgical intervention. Group 1 = patients who had glaucoma surgery prior to or concurrently with Kpro; Group 2 = patients with no history of glaucoma surgery or surgery ≥3 months post-Kpro. P-values calculated with Mann-Whitney *U* nonparametric t-test. n denotes number of eyes. Error bars represent Standard Error of the Mean. Statistical significance is defined as p < 0.05.

Preoperative best corrected visual acuity (BCVA) ranged from 20/400 to HM for both groups (with the median being CF in both), with an average LogMAR of 1.76 ± 0.26 for Group 1 and 1.81 ± 0.27 for Group 2 (p = 0.65). All patients had retained or improved BCVA within 3 months after BKPro (median 20/150 in Group 1 and 20/400 in group 2). Mean BCVA before and after BKPro at several time points are displayed in Fig 1. BCVA was retained better in Group 1 throughout the post-operatively course, but with only the 12-month timepoint reaching statistical significance (p = 0.028).

Loss of vision, as defined by the difference in LogMAR between best corrected vision after stabilization within 12 months and best corrected vision at 12, 24, and 36 months post-BKPro are shown in Table 2. Average BCVA within 12 months was 20/100 for Group 1 (range 20/30 to count fingers) and 20/140 (range 20/25 to HM) for Group 2. At the 12 months post-operative visits, vision loss in Group 1 was statistically significantly lower compared to Group 2 (p = 0.027), and at later follow-ups Group 1 had consistently lower BCVA loss, however there is no statistical difference.

**Table 2:** Vision loss, as determined by LogMAR of BCVA at several time points minus BCVA within 12 months post-Kpro, for patients with or without prior or concurrent glaucoma surgical intervention.

Vision Loss from Post-KPro BCVA within 12 months			
	Group 1 LogMAR ± SD, (n)	Group 2 LogMAR ± SD, (n)	p-value*
post-Kpro 12 months	-0.03 ± 0.02 (12)	-0.69 ± 0.36 (6)	0.027
post-Kpro 24 months	-0.10 ± 0.04 (10)	-0.54 ± 0.74 (6)	0.811
post-Kpro 36 months	-0.24 ± 0.07 (10)	-0.67 ± 0.99 (6)	1.000

*Mann-Whitney *U* nonparametric t-test used to compare mean BCVA between Group 1 and Group 2.

LogMAR = logarithm of minimum angle of resolution; SD = standard deviation; n = number of eyes.

Group 1 = patients who had glaucoma surgery prior to or concurrently with Kpro;

Group 2 = patients with no history of glaucoma surgery or 3 months post-Kpro.

Statistical significance is defined as p < 0.05.

**Fig 2:**
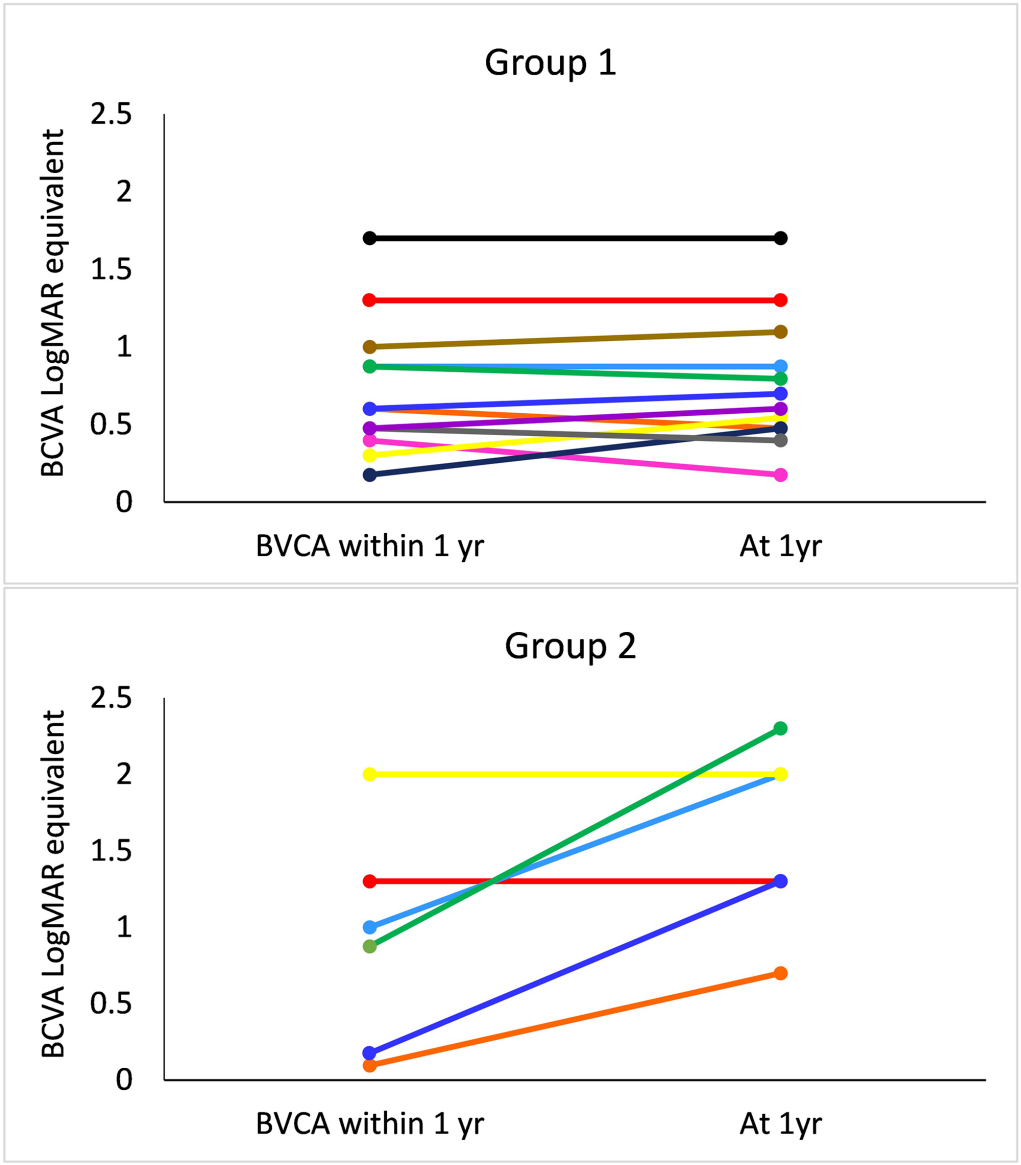
Best corrected visual acuity (BCVA) for patients with or without prior or concurrent glaucoma surgical intervention within 12 months and at 12 months post-Kpro implantation. Individual lines on graphs correspond to individual eyes. Group 1 = patients who had glaucoma surgery prior to or concurrently with Kpro; Group 2 = patients with no history of glaucoma surgery or 3 months post-Kpro.

Breakdowns of BCVA by each individual eye within 1 year post-BKPro and then at 1 year are shown in Fig 2. Four out of six eyes in Group 2 had significant worsening of vision at 1 year, and the two eyes that retained their BCVA had unchanged BCVA of 20/400 and HM within 1 year. Five out of 12 eyes in Group 1 had decreased BCVA, three had unchanged BCVA, and four had improved BCVA.

## Discussion

The use of the BKPro to restore vision in corneal blindness has been a significant milestone in therapy to offer to patients who previously had severely limited options for vision restoration. Although initial adoption of this procedure was slow, it has been more frequently offered in the last decade as surgeons have encountered better outcomes. Much of this success in improving outcomes using the BKPro procedure can be attributed to improved management of the most common post-operative issues [7–9]. Retroprosthetic membranes, which can be recurrent, are treated successfully with YAG laser [1]. The rate of post-operative endophthalmitis has been reduced with ongoing prophylactic topical antibiotic use [2]. Vision loss due to post-operative uncontrolled eye pressure and glaucoma is still a problematic post-operative issue for which a consensus approach is not widely used. Studying glaucoma in this population is highly challenging due to inability to visualize the optic nerve before or after surgery in many cases, and inability to test optic nerve function or structure. Furthermore, there is no standardized or reliable method for measuring IOP after BKPro surgery beyond digital estimation.

This retrospective review demonstrates better visual acuity retention one-year post operatively in patients who undergo glaucoma surgical intervention concurrently or close to the time of BKPro surgery. What is also of equal importance is the incidence of significant vision loss in the group of patients undergoing BKPro without close or concurrent glaucoma surgery. Although there are limitations in the outcome measures and it is difficult to accurately ascertain all causes of BCVA loss following BKPro, these results may suggest a trend of worsening vision loss over time when glaucoma is not prophylactically addressed at the time of BKPro surgery.

Surgeons have reached a consensus in measures to reduce the impact of retroprosthetic membranes and endophthalmitis after BKPro. Perhaps anticipating the need for glaucoma surgery at the time of BKPro can reduce the impact of vision loss from glaucoma post operatively. The way in which glaucoma surgery can be offered with BKPro requires careful consideration. Given the limitations in visualizing the peripheral anterior chamber after BKPro and the higher rates of postoperative infection, standard approaches to external filtration surgery are difficult. A trabeculectomy bleb may increase the lifetime risk of infection in an eye already prone to endophthalmitis, and placement of a tube into the anterior chamber or sulcus is precluded without the necessary visualization. In the past, authors have employed cyclodestructive procedures to be used in conjunction with the BKPro surgery with some success [10]. However, the inflammatory nature of cyclodestruction can possibly lead to rejection of a corneal graft and, in some cases, lead to cystoid macular edema that can result in reduced BCVA. In our review, the most common approach to glaucoma surgery at the time of BKPro was to use a glaucoma implant with a pars plana insertion of the tube. Tube placement was made possible by concurrent pars plana vitrectomy. This approach may offer the most predictable result in pressure lowering while reducing the possible post-operative adverse events [11].

Even in patients who have successful coordination of glaucoma surgery at the time of BKPro with stable BCVA over time, glaucoma has the potential to worsen and present as a later complication to BCVA. Digital applanation, although a crude method even with expert examiners, still offers some ability to gauge IOP when compared with the patient’s contralateral eye where Goldmann applanation may be possible. In patients where BCVA and cooperation allows serial visual field testing and optic nerve structural analysis, these tools may allow for close monitoring and escalation of treatment as needed. In patients where these tools are not possible, serial examination of the optic nerve head and comparison with baseline optic nerve photos may be a useful tool. If IOP control is in doubt, escalation of treatment with topical glaucoma medications should be strongly considered.

There are numerous limitations to this study. Firstly, there is no way to directly identify glaucoma as the main cause of vision loss in this group of subjects. While vision loss may be secondary to numerous contributing causes, our study does correlate early surgical intervention with better retention of vision. Secondly, this is a retrospective study with all the inherent limitations therein. Also, the number of subjects is limited, particularly in the late-intervention group. With a small sample size, adjustments based on age, gender, race, baseline IOP, or comorbidities would not yield meaningful conclusions. We also acknowledge that since visualization of the retina, optic nerve head, and measurement of IOP are challenging in these patients, comorbidities and complications such as retinal pathologies, suprachoroidal hemorrhage, and hypotony maculopathy cannot be consistently elicited and reliably gathered from all patient’s charts. These factors may confound visual acuity post-operatively. Lastly, selection bias may exist for patients with worse glaucomatous disease at baseline to undergo concurrent intervention. If such bias confounds our findings, however, they would likely further strengthen the relationship between early intervention and improved visual acuity outcomes.

In summary, we observed increased vision retention at 12 months post-op for patients who underwent early or concurrent glaucoma surgery with BKPro compared to patients who received late or no glaucoma surgery. Our findings suggest that prophylactic glaucoma surgery may be beneficial for patients undergoing K-pro implantation. Larger-scale prospective studies are needed to confirm the strength of this relationship and further characterize the complication profiles versus clinical benefit of concurrent glaucoma surgery with K-pro.

## Supporting information

S1 Table Original dataset containing matrix of patient’s visual acuity and intraocular pressure measurements at various timepoints. (XLSX)

## References

[1] Rudnisky CJ, Belin MW, Todani A, Al-Arfaj K, Ament JD, Zerbe BJ, et al Risk factors for the development of retroprosthetic membranes with Boston keratoprosthesis type 1: multicenter study results. Ophthalmology. United States; 2012;119: 951–955. 10.1016/j.ophtha.2011.11.030 PMC3343212 22361316

[2] Khan BF, Harissi-Dagher M, Khan DM, Dohlman CH. Advances in Boston Keratoprosthesis: Enhancing Retention and Prevention of Infection and Inflammation. Int Ophthalmol Clin. 2007;47(2). 10.1097/IIO.0b013e318036bd8b 17450007

[3] Banitt M. Evaluation and management of glaucoma after keratoprosthesis. Curr Opin Ophthalmol. United States; 2011;22: 133–136. 10.1097/ICU.0b013e328343723d 21191292

[4] Talajic JC, Agoumi Y, Gagne S, Moussally K, Harissi-Dagher M. Prevalence, progression, and impact of glaucoma on vision after Boston type 1 keratoprosthesis surgery. Am J Ophthalmol. United States; 2012;153: 267–274.e1. 10.1016/j.ajo.2011.07.022 21982110

[5] Robert M-C, Pomerleau V, Harissi-Dagher M. Complications associated with Boston keratoprosthesis type 1 and glaucoma drainage devices. Br J Ophthalmol. England; 2013;97: 573–577. 10.1136/bjophthalmol-2012-302770 23435225

[6] Panarelli JF, Ko A, Sidoti PA, Garcia JP, Banitt MR. Angle closure after Boston keratoprosthesis. J Glaucoma. United States; 2013;22: 725–729. 10.1097/IJG.0b013e318259b2fc 22595935

[7] Aldave AJ, Kamal KM, Vo RC, Yu F. The Boston Type I Keratoprosthesis. Improving Outcomes and Expanding Indications. Ophthalmology. American Academy of Ophthalmology; 2009;116: 640–651. 10.1016/j.ophtha.2008.12.058 19243830

[8] Bradley JC, Hernandez EG, Schwab IR, Mannis MJ. Boston type 1 keratoprosthesis: the university of california davis experience. Cornea. United States; 2009;28: 321–327. 10.1097/ICO.0b013e31818b8bfa 19387235

[9] Chew HF, Ayres BD, Hammersmith KM, Rapuano CJ, Laibson PR, Myers JS, et al Boston keratoprosthesis outcomes and complications. Cornea. United States; 2009;28: 989–996. 10.1097/ICO.0b013e3181a186dc 19724214

[10] Rivier D, Paula JS, Kim E, Dohlman CH, Grosskreutz CL. Glaucoma and keratoprosthesis surgery: role of adjunctive cyclophotocoagulation. J Glaucoma. United States; 2009;18: 321–324. 10.1097/IJG.0b013e3181815485 19365199

[11] Lenis TL, Chiu SY, Law SK, Yu F, Aldave AJ. Safety of Concurrent Boston Type I Keratoprosthesis and Glaucoma Drainage Device Implantation. Ophthalmology. American Academy of Ophthalmology; 2017;124: 12–19. 10.1016/j.ophtha.2016.08.003 27614591

